# Knowledge, attitudes, beliefs, values, preferences, and feasibility in relation to the use of injection safety devices in healthcare settings: a systematic review

**DOI:** 10.1186/s12955-016-0505-8

**Published:** 2016-07-13

**Authors:** Rami Tarabay, Rola El Rassi, Abeer Dakik, Alain Harb, Rami A. Ballout, Batoul Diab, Selma Khamassi, Elie A. Akl

**Affiliations:** Lebanese University, Beirut, Lebanon; American University of Beirut, Beirut, Lebanon; Faculty of Medicine, American University of Beirut, Beirut, Lebanon; World Health Organization, Geneva, Switzerland; Department of Internal Medicine, American University of Beirut, Beirut, Lebanon; Department of Internal Medicine, American University of Beirut Medical Center, P.O. Box: 11-0236, Riad-El-Solh Beirut, 1107 2020 Beirut Lebanon; Department of Clinical Epidemiology and Biostatistics, McMaster University, Hamilton, ON Canada

**Keywords:** HCW, Injection safety devices, Injections, Acceptance, Satisfaction, Preferences

## Abstract

**Background:**

Adopting technologies such as injection safety devices in healthcare settings can enhance injection safety. Developing guidelines for appropriate adoption of such technologies need to consider factors beyond evidence for their health effects. The objective of this study is to systematically review the published literature for evidence among healthcare workers and patients about knowledge, attitudes, beliefs, values, preferences, and feasibility in relation to the use of injection safety devices in healthcare settings.

**Methods:**

We included both qualitative and quantitative studies conducted with the general public, patients, and healthcare workers, administrators, or policy makers. We searched MEDLINE, EMBASE, CINHAL and CENTRAL. We used a duplicate and independent approach to title and abstract screening, full text screening, data abstraction and risk of bias assessment.

**Results:**

Out of a total of 6568 identified citations, we judged fourteen studies as eligible for this systematic review. All these studies were surveys, conducted with healthcare workers in high-income countries. We did not identify any qualitative study, or a study of the general public, patients, healthcare administrators or policy makers. We did not identify any study assessing knowledge, or values assigned to outcomes relevant to injection safety devices. Each of the included studies suffered from methodological limitations, which lowers our confidence in their findings. Based on the findings of six studies, the injection safety devices were generally perceived as easy to use and as an improvement compared with conventional syringes. Some of these studies reported few technical problems while using the devices. In three studies assessing perceived safety, the majority of participants judged the devices as safe. Two studies reported positive perceptions of healthcare workers regarding patient tolerance of these injection safety devices. One study found that less than half the nurses felt comfortable using the insulin pens. Findings from four studies assessing preference and satisfaction were not consistent.

**Conclusions:**

This systematic review identified evidence that injection safety devices are generally perceived as easy to use, safe, and tolerated by patients. There were few reports of technical problems while using the devices and some discomfort by nurses using the insulin pens.

**Electronic supplementary material:**

The online version of this article (doi:10.1186/s12955-016-0505-8) contains supplementary material, which is available to authorized users.

## Background

According to the World Health Organisation (WHO), there were 3 million exposures amongst healthcare workers (HCWs) in 2002 to blood borne pathogens due to needlestick injuries (NSI) [1]. The major pathogens of concern are hepatitis B virus (HBV), hepatitis C virus (HCV), and human immunodeficiency virus (HIV). Amongst HCWs, it is estimated that 37 % of Hepatitis B virus (HBV) infections, 39 % of Hepatitis C virus (HCV) infections, and 4.4 % of Human Immunodeficiency virus (HIV) infections are due to needlestick injuries [2]. NSI have also the potential to transmit other infectious agents, such as viral hemorrhagic fever viruses. Similarly, in the year 2000, the reuse of injection equipment accounted for 32, 40, and 5 % of new HBV, HCV, and HIV infections worldwide [1]. These infections will lead to a burden of 9.18 million disability-adjusted life years (DALYs) between the years 2000 and 2030 [3].

Adopting injection safety devices such as sharp injury protection (SIP) devices and reuse prevention (RUP) devices can enhance injection safety. The first stage of introducing an injection safety device into the clinical setting however, is an assessment of user acceptability [4]. These evaluations are usually conducted within a short timeframe and provide valuable information regarding user’s preferences and product characteristics [5]. Unfortunately, policies and guidelines for injection safety practices lack for a number of countries.

We conducted this study in preparation for the development of WHO policy guidance on use of injection safety devices by healthcare workers. The development of WHO policy guidance follows the GRADE methodology [6, 7]. The methodology calls for the consideration of factors beyond evidence for their health effects, including the valuation of the outcomes of interest, the preference for the different management options, their feasibility, and their effect on equity [6]. The consideration of these factors should be ideally based on a systematic review of the available evidence.

The objective of this study is to systematically review the published literature for evidence among healthcare workers and patients about knowledge, attitudes, beliefs, values, preferences, and feasibility in relation to the use of injection safety devices in healthcare settings.

## Methods

In this article, the term injection broadly refers to the use of sharp device in delivering skin injection or venous and arterial access for medication delivery and phlebotomy. We developed a protocol for this systematic review and registered it with PROSPERO [8].

### Eligibility criteria

#### Types of study designs

We included the following types of study designs:

- Quantitative studies including surveys
- Qualitative studies including individual interviews, and focus groups.
- Other study designs that specifically assess values and preferences including: time trade-off, probability trade-off, treatment trade-off, standard gamble, and visual analogue scales.
- Studies examining choices made when presented with decision aids
- Decision analyses

We excluded scientific meeting abstracts and research letters.

#### Types of study participants

We included studies conducted with the following types of participants:

- General public
- Patients with or without history of blood-borne infections due to injection in healthcare settings
- Healthcare workers with or without history of needle stick injury
- Healthcare administrators
- Healthcare policy makers (including those in the health insurance industry)

#### Types of settings

We included studies about the use of injection safety devices in the healthcare setting, i.e., healthcare workers delivering injections and patients receiving those injections for any reason (i.e., preventive, therapeutic, family planning). We were not interested in studies conducted in non-healthcare settings (e.g., illicit drug use, patients using insulin pen needles at home).

#### Types of injection devices

We were interested in both sharp injury protection devices and/or reuse prevention devices. We included studies assessing intramuscular (IM), subcutaneous (SC), intradermal (ID), phlebotomy and intravenous (IV)injections. Eligible injection safety devices included:

- Retractable needle device
- Needle shields, and recapping devices
- Needleless injectors
- Needle-safety devices
- Devices used for reconstitution methods which have a needle shield
- Auto-disable syringes (earlier called “auto-destruct syringes”), typically meant for vaccination
- Reuse prevention devices
- Pre-filled devices with reuse prevention feature
- Intravenous devices with a needle protection attribute
- IV catheters with a needle protection attribute
- All blood collection devices (lancet devices, vacuum tubes for blood collection devices, an arterial blood syringes).

We excluded studies using devices with needle protection attribute for articular, intra cardiac, and intra peritoneal injections.

#### Types of outcome measures

We included studies that assessed the following outcomes:

- Knowledge related to use of injection safety devices in healthcare settings;
- Attitudes and beliefs towards use of injection safety devices in healthcare settings;
- Values assigned to outcomes relevant to use of injection safety devices in healthcare settings (e.g., HIV infection). In other words, how much do those affected value the outcome?
- Preferences regarding the use of injection safety devices in healthcare settings;
- Feasibility of use of injection safety devices in healthcare settings. In other words, is it feasible to sustain the use of the device and to address potential barriers to implementing it?

### Literature search

We electronically searched (from date of inception to October 2013), the following databases used the OVID interface: MEDLINE, EMBASE, CINHAL and the Cochrane Central Register of Controlled Trials (CENTRAL). We did not use language or date restrictions. Additional file 1 lists the search strategy used in Medline. We also reviewed the references lists of relevant papers, searched of personal files for both published and unpublished stud2es and contacted experts.

### Selection process

Two teams of two reviewers each screened titles and abstracts of identified citations in duplicate and independently. We obtained the full texts of citations judged by at least one reviewer as potentially eligible. We conducted calibration exercises prior to screening in order to clarify the process. The two review teams screened the full texts for eligibility using a duplicate and independent approach and a standardized and pilot tested form. They resolved disagreements by discussion or with the help of a third reviewer when needed.

### Data abstraction process

A third review team abstracted data from eligible studies in duplicate and independent manner for English articles. For one non-English article, one reviewer abstracted the data. They used a standardized and pilot tested data abstraction form with detailed instructions. They resolved disagreements by discussion or with the help of a third reviewer when needed.

The abstracted data items included:

- Funding and reported conflicts of interest.
- Methodology including: type of study, survey instrument, sampling frame, sampling method, recruitment method, and survey administration method.
- Methodological quality. As there is no widely accepted tool for assessing the methodological quality of surveys, we abstracted information about the following: sample size calculation, sampling type, validity of tool, pilot testing, response rate, and handling of missing data.
- Population including: type, sample size, age, and gender.
- Setting: including the country.
- Device
- Outcomes assessed
- Results

### Data synthesis

We planned to report the results separately for two groups of devices: (1) intramuscular, subcutaneous, and intradermal injections; and (2) phlebotomy, and intravenous devices. This separation reflected the WHO expert panel decision to address these types separately in relation to their use in the clinical setting. We reported the statistical results for each study separately. We planned on stratifying results based on the type of participants/population (e.g., healthcare workers, patients) and based on the type of outcome assessed.

## Results

Please note that the use and mentioning of trade names in this article represents no endorsement of or advertisement for any product. The use of trade names was unavoidable as no generic names were identified for the devices evaluated here.

### Study selection

**Fig. 1:**
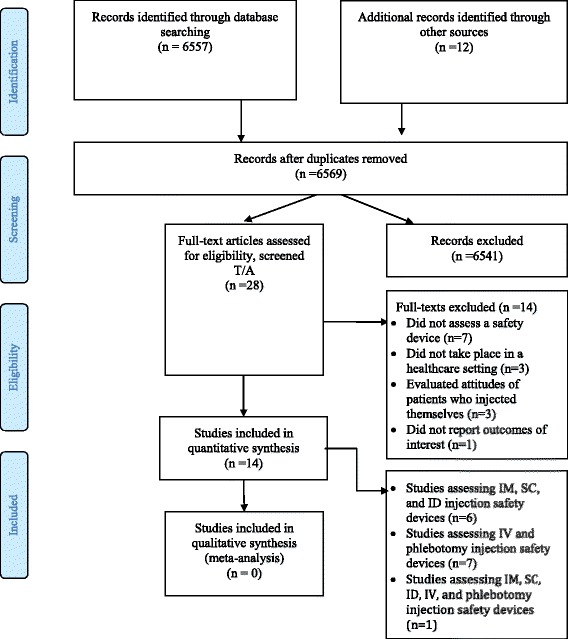
Study flow diagram

Figure 1 shows the study flow. Out of a total of 6569 identified citations, we judged fourteen as eligible for this systematic review [4, 9–21]. Of these, six studies reported data specific to subcutaneous, intramuscular, or intradermal injection safety devices [4, 9, 12, 13, 16, 17]. Seven studies reported data specific to intravenous or phlebotomy safety devices [10, 11, 14, 15, 18, 19, 21]. The fourteenth study reported data on any of the above types of devices [20].

Table 1 provides the list of the 14 excluded studies with the following reasons for exclusion: 7 of the studies did not assess an injection safety device [22–28], 3 studies were not conducted in a healthcare setting [29–31], 3studies reported attitudes of patients injecting themselves [32–34], and one study did not report on any of the outcomes of interest [35].

**Table 1:** List of excluded studies and reasons for exclusion

Study	Reason for exclusion
English, 1992 [22]	Study did not assess an injection safety device
Guerlain 2010 [29]	Study was not conducted in a health care setting
Hirayama 2009 [23]	Study did not assess an injection safety device
Jeanes 1999 [24]	Study did not assess an injection safety device
Kuroyama 2006 [25]	Study did not assess an injection safety device
Lee 2005 [35]	Study did not report on any of the outcomes of interest
Musso 2010 [32]	Study reported attitudes of patients injecting themselves
Oyer 2011 [26]	Study did not assess an injection safety device
Pfutzner 2013 [27]	Study did not assess an injection safety device
Quiroga1998 [30]	Study was not conducted in a health care setting
Shelmet 2004 [33]	Study reported attitudes of patients injecting themselves
Sibbitt 2008 [28]	Study did not assess an injection safety device
Tsu 2009 [31]	Study was not conducted in a health care setting
Vidovic 2010 [34]	Study reported attitudes of patients injecting themselves

### Study characteristics

Additional file 2 provides the characteristics of the included studies about subcutaneous, intramuscular, or intradermal injection safety devices. Additional file 3 provides the characteristics of the included studies about intravenous or phlebotomy safety devices.

#### Methodology

We did not identify any qualitative study. The included studies used a survey methodology using questionnaires. Four of these studies used 5-point or 10-point Likert scales [4, 9, 13, 16]. One study used a 7-point Likert scale [11]. There was poor reporting of the studies’ sampling frame, recruitment method, survey administration method, and sampling method. Vaudelle-Malbos et al. reported using convenience sampling, [20] Adams et al. reported a random selection of participants, [4], Adams et al. and Rivers et al. reported randomly selecting nurses [9, 19]. Prunette et al. reported cluster sampling [18].

#### Methodological quality

None of the studies reported sample size calculation. Three reported using a probability sampling approach [4, 9, 20]. Vaudelle-Malbos et al. reported non-probability sampling [20]. None of the other studies reported on sampling type. Davis et al. mentioned that the questionnaire used was based on “previously published validated and non-validated surveys” [12]. Butler et al. mentioned that the questionnaire used was adapted from a previously developed one for a new device questionnaire, and the validity was not reported [11]. Pilot testing was done in two studies [11, 19]. None of the remaining studies reported whether their questionnaires were pilot tested or validated. Eight studies reported on their response rates, which ranged from 5 % [20] to 100 % [4, 16]. None of the included studies reported on the handling of missing data. All these methodological limitations lower our confidence in the findings reported by those studies.

#### Devices

The devices assessed in the included studies were:

- Eclipse™, SafetyGlide™ and SafetyGlide™ insulin [4].
- SafetyGlide™hypodermic needles, SafetyGlide™-TNT (Tiny Needle Technology) insulin units and blunt fill cannulae [9].
- FlexPen®, and InnoLet® [12].
- Eclipse™, Magellan™, and SecureGard™ [13].
- Although the device brand was not reported, the injection safety device was described as having “a plastic sheath that extends over the needle tip to prevent accidental needle stick injuries. However, a spring-loaded plastic sheath covers the needle when a button is pushed with one finger. Thus, the safety feature is engaged with one hand after use” [16].
- Becton Dickinson 3 ml Safety-Lok™ syringe,and BaxterInterLink® needless intravenous system [17].
- Safety-Lok™, and Needle Pro®, Protectiv®I.V. catheter, and 2 needleless systems (Interlink® and Bionecteur®) [20].
- Needle-free system of all plastic material [15].
- Safety-Lok™, Punctur-Guard®, Venipuncture Needle-Pro® [22].
- BIO-SET®, double spike reconstruction device [29].
- Eclipse™, QuickShield® and Push Button™ [23].
- Introcan Safety® catheters, Insyte Autoguard™ [24].
- Protectiv® Plus IV [19].
- Clearlink ®needless connector [21].

#### Study funding

Of the seven included studies assessing subcutaneous, intramuscular, or intradermal injection safety devices, three reported their funding source, where two mentioned it as Becton Dickinson(Oxford, UK), [4, 9] and the other as Baxter Healthcare Corporation [17]. Of the seven included studies assessing intravenous and phlebotomy safety devices, three reported their funding source to be respectively: Department of Anesthesiology, Military Teaching Hospital Sainte Anne, Toulon, France; [18] Lamar University, Beaumont, Texas [19] Baxter Healthcare corporation [21].

#### Conflict of interest

One study reported that one author had conflict of interest with Novo.

Nordisk [12]. One study reported that their authors had no conflicts of interest [13]. The remaining studies did not provide conflicts of interest disclosures.

#### Population

Populations of thirteen studies included nursing populations, eight of which included physicians [4, 10, 14, 16–18, 20, 21]. One study did not describe its participant population [13]. The sample size of these studies ranged between 17 and 1705 participants. We did not identify any study of the general public, patients, healthcare administrators or policy makers.

#### Setting

Six of the studies were conducted in the United States [10, 11, 15–17, 19]. Two in France, [18, 20] Five in the United Kingdom, [4, 9, 13, 14, 21]. One study did not specify the country where it was conducted, but it appears to have been conducted in the United States [12]. All studies were conducted in the healthcare setting. We did not identify any study conducted in a low or middle-income country.

#### Outcomes assessed

The outcomes assessed included the following: perceived ease of use, perceived safety, perceived patient tolerance, perceived compatibility, reliability, confidence, preference, and satisfaction. One study assessed the predictors of acceptance of the device [19]. We did not identify any study assessing knowledge, or assigned to outcomes relevant to injection safety devices.

### Findings

We present below a summary of the findings of the included studies in relation to the each of the assessed outcomes. We first report the results relevant to IM, SC and ID injections, based on seven of the fourteen studies [4, 9, 12, 13, 16, 17, 20]. We then report the results relevant to IV/Phlebotomy devices based on eight of the fourteen studies [10, 11, 14, 15, 18–21].

### Intramuscular, subcutaneous and intradermal safety injections devices

#### Perceived ease of use

Six studies assessed ease of use and reported the following findings: [4, 9, 12, 13, 17, 20]

- 3 ml Safety-Lok™ was considered simple to use, and not associated with significantly more operator time [17].
- Safety-Lok™ was perceived as very easy to use, but with minor difficulties concerning the insertion of needle and obligation of one attempt [20].
- Between 70 % and 80 % of nurses agreed that FlexPen® and InnoLet® insulin pens were more convenient, simple and easy to use, and were an overall improvement compared with conventional vials/syringes [12].
- Most respondents felt that the SecureGard™ device required both hands to activate it [13]. One participant did not like putting the thumb near the needle tip to activate the Eclipse™ device, and other users made similar points about putting fingers near the tip of the Magellan™ device during activation [13].
- Eclipse™, SafetyGlide™ and SafetyGlide™ insulin were on average perceived as easy to activate, intuitive to use, did not hinder routine use, did not require more time to use than conventional products and did not require detailed training to use [4].
- In the study assessing SafetyGlide™ needles, SafetyGlide™-TNT insulin units, staff considered that the devices to be usable and compatible with most clinical situations [9].

#### Perceived safety

Three studies assessed perceived safety and reported the following findings: [4, 9, 20]

- Safety-Lok™, and Needle Pro® devices were perceived as safe. On third of users thought that the Needle-Pro device was not effective in avoiding needlestick injuries and therefore should not be implemented as a injection safety device [20].
- Eclipse™, SafetyGlide™ and SafetyGlide™ insulin were perceived to meet the safety standard, allowed activation to be clearly designated, could not be deactivated when reasonable force was applied [4].
- In the study assessing SafetyGlide™ needles, SafetyGlide™-TNT insulin units, staff considered that the devices to be safe [9].

#### Perceived patient tolerance

Two of the studies assessed patient tolerance towards the injection safety devices used and reported the following findings: [13, 16]

- The “safety syringes” (described as having a plastic sheath that extends over the needle tip) were perceived as comfortable [16].
- Eclipse™, Magellan™ and the SecureGard™ devices were not more painful to the patient compared to conventional devices [13].

#### Perceived compatibility

In one study, the safety feature of Eclipse™, SafetyGlide™ and SafetyGlide™ insulin were not perceived, on average, to hinder the product’s use. Only 6 % of nurses were concerned whether devices could be used for phlebotomy [4].

#### Reliability

Different users raised a number of reliability issues regarding the SecureGard™ product (e.g., leakage on one occasion, accidental activation of the device, needles falling off, safety feature not activating accurately on every occasion) [13].

#### Confidence

In the study assessing FlexPen® and InnoLet® insulin pens, 45 % of nurses agreed that they felt more comfortable giving insulin, using the pens compared with the conventional method [12]. Thirty nine percent of nurses agreed that they felt more confident they were giving the correct dose of insulin using pens compared with the conventional method [12].

#### Preference and satisfaction

Four studies assessed perceived preference or satisfaction of HCWs regarding the use of the devices and reported the following findings: [12, 13, 16, 17]

- Positive findings for FlexPen® and InnoLet® insulin pen devices [12]. Specifically, the nurses agreed that insulin pens were more convenient and that it took less time to prepare and administer insulin;
- Negative findings for the 3 ml Safety-Lok™ syringe. This was reportedly related to difficulty in engaging the protective sheath and because its size after being sheathed impeded easy disposal in needle boxes; [17].
- In one study, the shielded devices (Magellan™ and Eclipse™) were preferred over the retractable devices (SecureGard™), with Magellan being favored by most users [13].
- The overall satisfaction with the unspecified “safety syringe” was unfavorable, with lower median responses for post-use questions [16].

### Phlebotomy and intravenous injections devices

#### Perceived ease of use

Four studies [10, 14, 15, 18] assessed ease of use and reported the following findings:

- Seventy three percent said that Safety-Lok™, Punctur-Guard™, Venipuncture Needle-Pro were easier to use than the conventional device. Fifty eight percent said they facilitated the procedure, 31 % said they made the procedure more difficult, and 10 % were unsure [10].
- Concerning the Eclipse™, QuickShield® and Push Button™ some users indicated that certain orientations make it uncomfortable to hold the devices and perform venipuncture, while others indicated that shields could visually obstruct the needle.
  Results from the evaluations of the Push Button™ set were positive, i.e., the device was perceived easy to use.
  Some users commented that they did not like the position of the shield on the QuickShield and others stated that it was bulky, cumbersome and difficult to engage [14].
- Intensive care and critical care staff felt that the all-plastic material IV needle free system (anonymous brand) required too much manipulation and was too time consuming. Personnel were concerned that needle-free system would limit the fast flow rates during surgery [15].
- The passive Introcan® safety catheter was more efficient than the active Insyte Autoguard® safety catheter with regard to the ease of introducing the catheter into the vein and the staff’s exposure to the patient’s blood.
  The withdrawal of the needle was more difficult in the passive Introcan® Safety device group compared with the Vialon® and the active Insyte Autoguard® groups [19].
- A positive response (measured as ease of use) was given of more than 85 %, regarding the usability of the needless connector [21].

#### Perceived safety

Four studies [10, 18–20] assessed perceived safety and reported the following findings:

- The catheter Protectiv® was not safe to use [20].
- Sixty seven percent reported that the vacuum tube blood-collection needle with recapping sheath made the procedures safer to perform, 52 % reported that the bluntable vacuum tube blood-collection needle (Venipuncture Needle-Pro®) was safe, and 56 % reported that the resheathable winged steel needle device (Safety-Lok™) was safe [10].
- Ninety two percent agreed that proper use of the device protects from needlestick injury [19].
- The staff’s sense of protection against the risk of an accidental needlestick was equal and more favorable with the safety catheters (passive safety Introcan® Safety catheters, active safety Insyte Autoguard®) than with the conventional catheter (Vialon®, the nonsafety IV catheter) [18].
  1. In the study assessing Clearlink®needless connector, 70 % preferred to use the safer sharps device rather than a conventionally used luer cap [21].

#### Perceived compatibility

Three studies [14, 15, 21] assessed perceived safety and reported the following findings:

- Compared with the Eclipse™, fewer users felt that the QuickShield® should be considered for use in the Welsh NHS or would consider using it instead of a conventionally used blood evacuation needle [14].
- Eighty nine percent of the nurses thought that the needle free all-plastic material IV system (brand not specified) was the answer to the IV-related needle punctures [15].
- Eighty five percent of health care workers considered the Clearlink®needless connector to be suitable for every day practice [21].

#### Confidence

Three studies assessed the confidence perceived using the IV injection safety devices: [14, 19, 21].

In the study assessing Eclipse™, QuickShield® and Push Button™, more users appeared to be comfortable using the Eclipse™ than the QuickShield. Most users found the Push Button™ set comfortable to use [14].

Eighty six percent of nurses mentioned that they will always use Protectiv Plus® IV device where eighty three percent felt comfortable using Protectiv Plus® IV [19].

In the study assessing the needless connector, 85 % of health care workers felt confident to use the device after caring for 3 patients [21].

#### Reliability

Eight percent reported having difficulty to avoid leak of blood after removal of needle from the vein using the Protectiv® IV catheter [20].

In the study assessing the needless connector Clearlink®, only 1respondent noted a tendency for the device, to ‘pop off’ when used with a luer lock syringe [21].

#### Predictors of acceptance

In the study assessing the Protectiv Plus® IV [19], they assessed the individual and organizational predictors of acceptance of the device by nurses. Almost half of the nurses (48.6 %) had used the device more than 12 months. The majority of nurses (69.6 %) agreed to the appropriateness of time between training and use of device. The majority (76.9 %) disagreed that they needed more time for training. The authors mentioned that a positive safety climate existed, referring specifically the fast training suggesting considerable acceptance of the device between nurses [19].

#### Preference and satisfaction

Five studies [10, 11, 14, 15, 19] assessed perceived preference or satisfaction of HCWs regarding the use of the devices and reported the following findings:

- Fifty seven percent favored phlebotomy needle with recapping sheath (Safety-Lok™), 26 % favored bluntable phlebotomy needle (Punctur-Guard®), and 47 % favored resheathable winged steel needle compared with the respective conventional devices. Twenty three percent of respondents had no preference [10].
  1. Nurses scored the needless device Bioset almost twice as high as conventional method non safety on total preference [11].
- The Eclipse product was favored slightly more than the QuickShield (the two being phlebotomy safety devices) [14].
- Ninety five percent of the nurses responded that an unspecified IV system was preferred to other needle-free systems evaluated [15].
- Seventy six percent of nurses agreed that they are generally satisfied with the Protectiv-R Plus® IV device [19].

## Discussion

In summary, this systematic review identified evidence from fourteen studies suggesting that injection safety devices are generally perceived as easy to use, safe, and tolerated by patients. There were few reports of technical problems while using the devices and some discomfort by nurses using the insulin pens. Nurses’ preferences and satisfaction were not consistent in studies related to intramuscular, subcutaneous and intradermal injections. Unfortunately, the included studies suffered from methodological limitations, which lowers our confidence in their findings.

In one study, the appropriate use of an injection safety device with “a plastic sheath that extends over the needle tip” was reported to be suboptimal (at most 60 %), even after an educational presentation [16]. That same paper described the challenge of activating the safety feature through additional steps that require time, effort, and depend on whether the user remembers to perform the task. In another study, the authors described the passive Introcan safety catheter as more efficient than the active Insyte Autoguard safety catheter with regard to the ease of introducing the catheter into the vein and the staff’s exposure to the patient’s blood [16]. These findings highlight the importance of educational intervention, buy-in, and use of simplified devices, such as passive rather than active injection safety devices [16].

This study has a number of limitations and strengths. The limitations are mainly related to the small number and low quality of the available evidence. In addition, we did not identify any qualitative study and none of the included studies was conducted in low or middle-income countries or included the general public, patients, healthcare administrators or policy makers. We did not identify any study assessing knowledge, or values assigned to outcomes relevant to injection safety devices. It is important to note that we did not include studies relevant to articular, intra cardiac, and intra peritoneal injection devices. The main strength of this study relates to the use of standard approaches in conducting [36], and reporting systematic reviews [37].

We have identified one other systematic review, published by the Health and Safety Laboratory for the Health and Safety Executive 2012, [38] addressing whether the use of injury prevention devices in healthcare affect user acceptability compared to standard practices. The results of that review were consistent with ours and they concluded that “health care workers should be involved in the evaluation of products before any safer sharps device is introduced” and that “user acceptability is likely to be an obstacle to the uptake of safer sharps devices”. That review included only five studies potentially relevant to our question, all of which we have identified and included in this review [4, 10, 18, 19, 21]. They included two studies that we think are not eligible for our review. They missed to include nine other studies that we judged as eligible and have included in our review [9, 11–17, 20].

## Conclusions

Our findings have implications for clinical practice. Healthcare workers perceive injection safety devices to be generally easy to use, safe, and tolerated by patients. It is not clear whether some of the reported technical problems, and the extra steps required for using the injection safety devices explain the inconsistency between the included studies in preferences and satisfaction. There is a need to engage healthcare workers in the selection of the injection safety devices to ensure user acceptability and the eventual desired beneficial effect on disease burden.

Our findings have also research implications. Future research should include qualitative studies to better understand the findings above. There is also a need to conduct studies low or middle-income countries, and studies assessing knowledge, and values assigned to outcomes relevant to injection safety.

## Additional files

Additional file 1: Search strategy used in Medline. (DOC 29 kb) Additional file 2: Characteristics of the included studies on subcutaneous, intramuscular, or intradermal injection safety devices. (DOC 79 kb) Additional file 3: Characteristics of the included studies on intravenous and/or phlebotomy injection safety devices. (DOCX 37 kb)
