# Supplementary material for: Knowledge, attitudes, beliefs, values, preferences, and feasibility in relation to the use of injection safety devices in healthcare settings: a systematic review
Source: Health Qual Life Outcomes. 2016 Jul 13;14:102. doi: 10.1186/s12955-016-0505-8 (PMC4944234; doi:10.1186/s12955-016-0505-8)
Supplement: Additional file 1: — Search strategy used in Medline. (DOC 29 kb) [file 12955_2016_505_MOESM1_ESM.doc]

**Additional file 1:** Search strategy used in Medline

1 Health Personnel/

2 Personnel, Hospital/

3 ((Healthcare or health-care or (health adj care)) adj2 worker*).mp.

4 Paramedic*.mp.

5 ((medical or nurs*or ancillary) adj2 staff*).mp.

6 (Medical adj2 laboratory adj2 techn*).mp.

7 Pharmacist*.mp.

8 physician*.mp.

9 Hospitalist*.mp.

10 internist*.mp.

11 doctor*.mp.

12 Phlebotomist*.mp.

13 exp Needlestick Injuries/

14 exp Accidents, Occupational/ and (syringe* or needle* or inject*).mp.

15 (injur* adj3 (syringe* or needle* or inject*)).mp.

16 exp Accidents, Occupational/ and (syringe* or needle* or inject*).mp.

17 exp Accident Prevention/ and (syringe* or needle* or inject*).mp.

18 (blood adj3 collection adj3 (syringe* or needle* or system* or device* or material* or product* or set*)).mp.

19 ((need-less or needless or needle-free or needlefree) adj3 (syringe* or needle* or system* or device* or material* or product* or set* or inject*)).mp.

20 (Single adj3 "use" adj3 (syringe* or needle* or system* or device* or material* or product* or set* or inject*)).mp.

21 (prevent* adj3 (syringe* or needle* or system* or device* or material* or product* or set* or inject*)).mp.

22 (reuse adj3 (syringe* or needle* or system* or device* or material* or product* or set* or inject*)).mp.

23 (exp Equipment Reuse/ or exp Disposable equipment/) and (syringe* or needle* or system* or device* or material* or product* or set* or inject*).mp.

24 (Disposable adj2 equipment* adj3 (syringe* or needle* or inject*)).mp.

25 ((prefill* or pre-fill*) adj3 (syringe* or needle* or inject*)).mp.

26 (Autopen or auto-pen).mp.

27 "Vetter Lyo-ject".mp.

28 Vasceze.mp.

29 Sterimatic.mp.

30 "Safe-Point".mp.

31 "Needle-Pro".mp.

32 Hypak.mp.

33 VanishPoint.mp.

34 "Slip-lock".mp.

35 Luerlok.mp.

36 "Bio-Set".mp.

37 "Auto-disposable syringe*".mp.

38 ((prefill* or pre-fill*) adj2 syringe*).mp.

39 "BD Hypak".mp.

40 "Safety-Lok".mp.

41 (Kendall's adj2 Monoject).mp.

42 "autodestruct syringe".mp.

43 SoloShot.mp.

44 "Monodose syringe*".mp.

45 "Unifine pentip*".mp.

46 Autoject.mp.

47 (ultrasafe adj passive adj delivery adj system).mp.

48 "Tip-Lok".mp.

49 "Gettig Guard".mp.

50 "Inviro SNAP!".mp.

51 "Maxxon safety syringe*".mp.

52 "monoject magellan".mp.

53 "needle-pro".mp.

54 "point-lok".mp.

55 "wandplus".mp.

56 "safetyglide".mp.

57 "safety wand".mp.

58 "powder ject".mp.

59 or/1-12

60 or/13-58

61 and/59-60
