# Supplementary material for: Knowledge, attitudes, beliefs, values, preferences, and feasibility in relation to the use of injection safety devices in healthcare settings: a systematic review
Source: Health Qual Life Outcomes. 2016 Jul 13;14:102. doi: 10.1186/s12955-016-0505-8 (PMC4944234; doi:10.1186/s12955-016-0505-8)
Supplement: Additional file 2: — Characteristics of the included studies on subcutaneous, intramuscular, or intradermal injection safety devices. (DOC 79 kb) [file 12955_2016_505_MOESM2_ESM.doc]

**Additional file 2:** Characteristics of the included studies on subcutaneous, intramuscular, or intradermal injection safety devices.

| **Study ID, Funding** | **Methodology** | **Methodological quality** | **Population/Setting** | **Devices** | **Outcomes and results** |
| --- | --- | --- | --- | --- | --- |
| Adams, 2003  Funding: Educational grant from Becton Dickinson  Conflict of interest not reported | - **Type of study**: survey - **Survey instrument**: a questionnaire with 10 statements scored on a Likert scale (1= strongly agree, and 5= strongly disagree), and additional specific questions about devices. - **Sampling frame**: not reported - **Sampling method**: randomly selected - **Recruitment method**: not reported - **Administration method**: not reported | - **Sample size calculation**: not reported - **Sampling type**: probability sampling - **Validity of tool**: standardized user evaluation questionnaire adapted from Emergency Care Research Institute (ECRI); no validation reported - **Pilot testing done**: not reported - **Response rate**: 100% - **Handling of missing data:** not reported | - **Population**: nurses from a range of specialties - **N**: 50 - **Age**: not reported - **Gender**: not reported - **Country**: UK - **Setting**: University Hospital Birmingham NHS Trust | - 3 needle-safety devices: Eclipse™, SafetyGlide™ and SafetyGlide™ insulin | - **Perceived safety**: the 3 devices met the safety standard (mean score range = 1.78-1.88), allowed activation to be clearly designated (mean score range = 1.30-1.58), could not be deactivated when reasonable force was applied (mean score range = 1.60-1.74). - **Perceived ease of use**: the 3 devices were easy to activate (mean score range = 1.58-1.70), intuitive to use (mean score range = 1.74-1.78), did not hinder routine use (mean score range = 1.70-1.2), did not require more time to use than conventional products (mean score range = 1.74-1.80) and did not require detailed training to use (mean score range = 1.60-1.74) - **Perceived compatibility**: the safety feature did not hinder the product’s use (mean score range = 1.70-2.1). Only 6% of nurses were concerned whether devices could be used for phlebotomy. |
| Adams 2006  Educational grant from Becton Dickinson (Oxford, UK)  Conflict of interest not reported | - **Type of study:** survey - **Survey instrument**: a questionnaire with 10 statements scored on a Likert scale (1= strongly agree, and 5= strongly disagree) after one, 6 and 11 months of introduction of devices - **Sampling frame**: nurses from four clinical areas at the University Hospital Birmingham NHS Foundation Trust; two surgical wards, one medical ward and one outpatient department. - **Sampling method**: randomly selected - **Recruitment method**: not reported - **Administration method**: not reported | - **Sample size calculation**: not reported - **Sampling type**: probability sampling - **Validity of tool**: user evaluation form, validity not reported - **Pilot testing done**: not reported - **Response rate**: not reported - **Handling of missing data:** not reported | - **Population**: healthcare workers from four clinical areas - **N**: 51 - **Age**: not reported - **Gender**: not reported - **Country**: UK - **Setting**: University Hospital Birmingham NHS Trust; two surgical wards, one medical ward and one outpatient department. | - 3 devices: SafetyGlide™ needles, SafetyGlide-TNT ™ insulin units and blunt fill cannulae | - **Perceived safety:** Staff considered that the devices were safe - **Perceived ease of use:** Staff considered that the devices were usable - **Perceived compatibility:** Staff considered that the devices were compatible with most clinical situations.   In particular, it was considered that the safety needle devices should be used for any procedure where a risk of exposure to blood and body fluids existed. |
| Davis, 2009 [10]  Funding: not reported  No conflict of interest reported | - **Type of study**: survey - **Survey instrument**: an investigator-developed survey tool asking nurses to answer satisfaction questions comparing insulin pen devices to vials/syringes - **Sampling frame**: not reported - **Sampling method**: not reported - **Recruitment method**: not reported - **Administration method**: not reported | - **Sample size calculation**: not reported - **Sampling type**: not reported - **Validity of tool**: survey designed based on previously published validated and non-validated surveys. - **Pilot testing done**: not reported - **Response rate**: 67% - **Handling of missing data:** not reported | - **Population**: registered nurses on 2 medical-surgical floors - **N**: 54 - **Age**: not reported - **Gender**: not reported - **Country**: not reported - **Setting**: community hospital | - 2 devices: insulin pen devices (FlexPen® and InnoLet®) compared with vial/syringes | - **Preferences**: 80% of nurses responded positively and agreed that insulin pens were more convenient, while 70% agreed that it took less time to prepare and administer insulin using pens compared with the conventional method.   Overall, 69% of nurses agreed that insulin pens were an improvement over the conventional method.  69% nurses agreed they were more satisfied with preparing insulin and 61% felt they were more satisfied administering insulin using pens compared with the conventional method.  **Perceived ease of use:** 70% to 80% of nurses agreed that FlexPen and InnoLet insulin pens were more convenient, simple and easy to use, and were an overall improvement compared with conventional vials/syringes.   - **Confidence**: 45% of nurses agreed that they felt more comfortable giving insulin, using pens compared with the conventional method. 39% of nurses agreed that they felt more confident they were giving the correct dose of insulin using pens compared with the conventional method. |
| Ford 2011 [11]  Funding: not reported  No conflict of interest reported. | - **Type of study**: survey - **Survey instrument**: a questionnaire evaluating all devices, then a final questionnaire scored using a likert scale between 1 and 5: 1 being strongly agree, 5 being strongly disagree. - **Sampling frame**: not reported - **Sampling method**: not reported - **Recruitment method**: not reported - **Administration method**: not reported | - **Sample size calculation**: not reported - **Sampling type**: not reported - **Validity of tool**: not reported - **Pilot testing done**: not reported - **Response rate**: not reported - **Handling of missing data:** not reported | - **Population**: participants - **N**: 61 (19 evaluated the Eclipse, 22 the Magellan and 20 the SecureGard) - **Age**: not reported - **Gender**: not reported - **Country**: UK - **Setting**: 5 Welsh NHS boards and 1 local health board | - 3 devices: the Eclipse™, the Magellan™ and the SecureGard™ | - **Perceived safety**: one participant commented that they did not like putting their thumb near the needle tip to activate the Eclipse device, and other users made similar points about putting fingers near the tip of the Magellan device during activation. - **Reliability**: different users raised a number of reliability issues regarding the SecureGard product. One participant reported leakage from the syringe on one occasion, another reported accidental activation of the device, two participants complained of needles falling off, and 5 stated that the safety feature did not activate accurately on every occasion. - **Perceived ease of use:** most respondents felt that the SecureGard device required both hands to activate it.   Two respondents described Eclipse as ‘cumbersome’ and one respondent described it as ‘bulky’. One user described the Magellan as ‘bulky’ by and another described it as ‘clumsy’.   - **Preferences:** the shielded devices (Magellan and Eclipse) were preferred over the retractable devices (SecureGard), with Magellan being favored by most users. - **Perceived patient tolerance:** none of the 3 devices was perceived to be more painful to the patient than conventional devices. |
| Mulherin 1996 [14]  Funding: not reported  Conflict of interest not reported | - **Type of study**: prospective survey, pre-use, post use matched survey - **Survey instrument**: a questionnaire administered after one month of use. Answers based on a Likert scale with 1=poor, 5=average and 10=excellent. - **Sampling frame**: not reported - **Sampling method**: not reported - **Recruitment method**: not reported - **Administration method**: not reported | - **Sample size calculation**: not reported - **Sampling type**: not reported - **Validity of tool**: no validation reported - **Pilot testing done**: not reported - **Response rate**: 100% - **Handling of missing data:** not reported | - **Population**: nurses (76.4%)/ HCWs at the emergency department - **N**: 17 - **Age**: not reported - **Gender**: not reported - **Country**: USA - **Setting**: University of California, San Diego Medical Center Hill-Crest, an urban tertiary-care medical center. | - A safety syringe that “has a plastic sheath that extends over the needle tip to prevent accidental needle stick injuries. However, a spring-loaded plastic sheath covers the needle when a button is pushed with one finger. Thus, the safety feature is engaged with one hand after use”. | - **Satisfaction:** on all aspects of the post-use questionnaire, except for patient safety and comfort, the overall satisfaction with the safety syringe was unfavorable (P<0.0186), with lower median responses for post-use questions. |
| Orenstein 1995  Funding: supported in part by a grant from Baxter Healthcare Corporation  Conflict of interest not reported | - **Type of study**: survey - **Survey instrument**: product evaluation questionnaires collected at 1 and 3 month - **Sampling frame**: not reported - **Sampling method**: not reported - **Recruitment method**: not reported - **Administration method**: not reported | - **Sample size calculation**: not reported - **Sampling type**: not reported - **Validity of tool**: not reported - **Pilot testing done**: not reported - **Response rate**: not reported\ - **Handling of missing data:** not reported | - **Population**: nursing personnel (registered nurses, licensed practical nurses, nurse aides, and nursing students) working on the study units and of house staff teams (attending and resident physicians, interns, and medical students) rotating through these units - **N**: 262 - **Age**: not reported - **Gender**: not reported - **Country**: USA - **Setting**: 6 hospital inpatient units; 3 medical units, 2 surgical units and 1 surgical-trauma intensive care unit at a 900-bed urban university medical center, Medical College of Virginia Hospital | - 2 devices: the Becton Dickinson 3ml Safety-Lok™ syringe and the Baxter InterLink®needless intravenous system | - Perceived ease of use: Both devices were considered simple to use, and not associated with significantly more operator time. - Preference: the 3 ml safety syringe was least favored device because of difficulty in engaging the protective sheath and because its size after being sheathed impeded its easy disposal in needle boxes.   Perceived Compatibility:  Only 73% of those surveyed supported purchasing the Safety- Lok syringes, versus 94% support for the Inter-Link needleless intravenous system components. |
| Vaudelle-Malbos, 1996  Funding: not reported  Conflict of interest not reported | - **Type of study**: survey - **Survey instrument**: questionnaire in two sections: first section about ease of use and safety of the device (included binary evaluation yes/no and a 6 point scale); and a second section that asked specific questions about devices. - **Sampling frame**: not reported - **Sampling method**: convenience sampling (departments selected based on utilization patterns) - **Recruitment method**: not reported - **Administration method**: not reported | - **Sample size calculation**: not reported - **Sampling type**: non-probability sampling - **Validity of tool**: questionnaire elaborated with the collaboration of the hygiene supervisor of each hospital; no validation reported - **Pilot testing done**: not reported - **Response rate:**   Safety-Lok: 37%  Needle-Pro: 35%  Protectiv: 89%  Interlink®: 86%  Bionecteur®: 40%   - **Handling of missing data:** not reported | - **Population**: HCWs (nurses in majority, radiology department, physicians) from a range of specialties - **Number of participants**: varied between 22 and 163 - **Age**: not reported - **Gender**: not reported - **Country**: France - **Setting**: Sainte-Anne hospital | 3 needle protection devices: Safety-Lok™, Needle Pro®, I.V. catheter Protectiv® and 2 needleless systems (Interlink® and Bionecteur®) | - **Perceived ease of use:** Safety-Lok was perceived as very easy to use, but with minor difficulties concerning the insertion of needle and obligation of one attempt.   **Reliability:** Catheter Protectiv: 8% reported having difficulty to avoid leak of blood after removal of needle from the vein    **Perceived safety:** all devices were perceived as safe except the catheter Protectiv  1/3 of users thought that the Needle-Pro device was not effective in avoiding needlestick injuries and therefore should not be implemented as a safety device. |
