# Supplementary material for: Knowledge, attitudes, beliefs, values, preferences, and feasibility in relation to the use of injection safety devices in healthcare settings: a systematic review
Source: Health Qual Life Outcomes. 2016 Jul 13;14:102. doi: 10.1186/s12955-016-0505-8 (PMC4944234; doi:10.1186/s12955-016-0505-8)
Supplement: Additional file 3: — Characteristics of the included studies on intravenous and/or phlebotomy injection safety devices. (DOCX 37 kb) [file 12955_2016_505_MOESM3_ESM.docx]

**Additional file 3:** Characteristics of the included studies on intravenous and/or phlebotomy injection safety devices.

| **Study ID, Funding** | **Methodology** | **Methodological quality** | **Population/Setting** | **Devices** | **Outcomes and results** |
| --- | --- | --- | --- | --- | --- |
| Alvarado-Ramy, 2003[[8](#_ENREF_1)]  Funding: not reported  Conflict of interest not reported | - **Type of study:**survey - **Survey instrument:**“device evaluation survey” - **Sampling frame:**not reported - **Sampling method:**not reported - **Recruitment method/ Administration method:**Questionnaires distributed at staff meetings, educational conferences, and nurses’ stations. At one hospital, questionnaires were mailed to medical students who were otherwise inaccessible. | - **Sample size calculation:**not reported - **Sampling type:** not reported - **Validity of tool:** no validation reported - **Pilot testing done:**not reported - **Response rate:**65% - **Handling of missing data:** not reported | - **Population:** healthcare workers - **N:**1705 - **Age**: not reported - **Gender**: not reported - **Country**: USA - **Setting:**10 university-affiliated hospitals in Minneapolis–St. Paul, Minnesota (3 hospitals), New York, New York (1 hospital), San Francisco, California (4 hospitals), and Houston, Texas (2 hospitals) | - 3 devices: resheathable winged steel needles (Safety-Lok™), bluntable vacuum tube blood-collection needles (Punctur-Guard®) and resheathable vacuum tube blood collectionneedles (Venipuncture Needle-Pro®) | - **Preference:** 44% favored one or more of the safety devices, 33% favored the conventional devices, 23% were unsure. 57% favored phlebotomy needle with recapping sheath, 26% favored bluntable phlebotomy needle, and 47% favored resheathable winged steel needle compared with the respective conventional devices. 23% of respondents had no preference. - **Perceived ease of use:**73% said the safety device was easier to use than the conventional device. 58% said it facilitated the procedure, 31% said it made the procedure more difficult, and 10% were unsure. - **Perceived safety:**“devices made the procedures safer to perform”: 67% for the vacuum tube blood-collection needle with recapping sheath, 52% for the bluntable vacuum tube blood-collection needle, and 56% for the resheathable winged steel needle device. |
| Butler, 2005[[9](#_ENREF_2)]  Funding: not reported  Conflict of interest not reported | - **Type of study:**cross-sectional study - **Survey instrument:**questionnaire scored on a 7-point Likert scales ranging from strongly agree to strongly disagree. - **Sampling frame:**not reported - **Sampling method:**not reported - **Recruitment method:** not reported - **Administration method:**survey questionnaire | - **Sample size calculation**: not reported - **Sampling type:**not reported - **Validity of tool:**adapted from a previously developed one for a new device questionnaire, no validity reported. - **Pilot testing done**: Yes - **Response rate:**not reported - **Handling of missing data:** not reported | - **Population:**nurses who infuse patients - **N:**59 - **Age**: 42.4 yrs - **Gender:**not reported - **Country:**USA - **Setting:**not reported | 2 devices: BIO-SET®, a needleless device compared with conventional method and a double spike reconstruction device for the preparation of recombinant factor VII. | - **Preferences:**Nurses scored the needless device almost twice as high as conventional method on total preference. |
| Ford,2011- blood evacuation  [[12](#_ENREF_4)]  Funding: not reported  Conflict of interest not reported | - **Type of study:**survey - **Survey instrument:**a questionnaire for each type of device, then a questionnaire comparing the two shielded evacuation needles. - **Sampling frame:**not reported - **Sampling method:**not reported - **Recruitment method:** not reported - **Administration method:**not reported | - **Sample size calculation:** not reported - **Sampling type**: not reported - **Validity of tool:**not reported - **Pilot testing done:**not reported - **Response rate:** not reported - **Handling of missing data:** not reported | - **Population:**Health care workers. - **N:**84 (33 evaluated the Eclipse, 30 the QuickShield and 21 the Push Button) - **Age**: not reported - **Gender:**not reported - **Country:**UK - **Setting:**7 Welsh NHS boards and the Welsh Blood Service | 3 devices: the Eclipse™, QuickShield® and Push Button™ devices following a blood taking procedure. | - **Preferences:**the Eclipse product was favored slightly more than the QuickShield®   **Perceived compatibility:**Compared with the Eclipse™, fewer users felt that the QuickShield® should be considered for use in the Welsh NHS or would consider using it instead of a conventional blood evacuation needle.  **Perceived ease of use:**Some users indicated that certain orientations make it uncomfortable to hold the device and perform venipuncture, while others indicated that shields could visually obstruct the needle. Results from the evaluations of the Push Button™ set were positive.Some users commented that they did not like the position of the shield on the QuickShield® and others stated that it was bulky, cumbersome and difficult to engage.  **Confidence:**More users appeared to be comfortable using the Eclipse™ than the QuickShield.®  Most users found the Push Button™ set comfortable to use. |
| Wolfrum 1994[[13](#_ENREF_7)]  Funding: not reported  Conflict of interest not reported | **Type of study:** survey  Survey instrument: not reported  **Sampling frame:** not reported  **Sampling method:** not reported  **Recruitment method:**not reported  **Administration method:** self-administered | **Sample size calculation**: not reported  **Sampling type:** not reported  **Validity of tool**: not reported  **Pilot testing done:**not reported  **Response rate:** 30%  **Handling of missing data:** not reported | **Population**: nurses  **N:** not reported  **Age:** not reported  **Gender**: not reported  **Country:** USA  **Setting:** a 394-bed acute-care hospital/teaching facility in Wisconsin | A needle-free system of all plastic material  . | **Preferences:** 95% of the nurses responded that the IV system was preferred to other needle-free systems evaluated.  **Perceived compatibility**: 89% of the nurses thought that the IV system was the answer to the IV-related needle punctures.  **Perceived ease of use**: Intensive care and critical care staff felt the system required too much manipulation and was too time consuming. OR personnel were concerned that needle-free system would limit the fast flowing rates during surgery. |
| Prunet, 2008[[1](#_ENREF_6)6]  Funding: Sainte Anne Hospital and Department of  Anesthesiology  Conflict of interest not reported | - **Type of study:** survey - **Survey instrument:**   A Visual Analog Scale (VAS), ranging from (0 to 10) to assess ease of use (very easy to very difficult) and perceived safety (very safe to not at all safe)   - **Sampling frame:** not reported - **Sampling method:** Cluster sampling (The patient characteristics were similar for the three - IV catheters) - **Recruitment method:** Not reported - **Administration method:** Not reported | **Sample size calculation:**not reported  **Sampling type:**Not reported   - **Validity of tool:** Not reported - **Pilot testing done:**Not reported - **Response rate**(for the 3 types of devices): 100% - **Handling of missing data:** not reported | - **Population:**The anesthetists–physicians and the anesthetists–nurses in the operating room, and the emergency physicians and the nurses in the emergency department - **Number of participants:**not reported - **Age:**Not reported - **Gender:**Not reported - **Country:**France - **Setting:**   Department of Anesthesiology, Military Teaching Hospital Sainte Anne | 3 peripheral IV catheters: Vialon™ (the nonsafety catheter), passive safety Introcan Safety® catheters, active safety Insyte Autoguard™ | - **Perceived ease of use:**Users found their usemore difficult and their handling generates more splashing of blood into the environment.The passive Introcansafety catheter was more efficient than the active Insyte Autoguard safety catheter with regard to the ease of introducing the catheter into the vein and the staff’s exposure to the patient’s blood.   The withdrawal of the needle was more difficult in the passive Introcan Safety® group compared with the Vialon and the active Insyte Autoguard™ groups.   - **Perceived safety:**   The staff’s sense of protection against the risk of an accidental needlestick was equal and more favorable with the safety catheters than with the conventional catheter. |
| Rivers, 2003[[17](#_ENREF_5)]  Funding: A Research Enhancement Grant from Lamar University, Beaumont,  Texas provided partial funding for the study.  Conflict of interest not reported | - **Type of study:**survey - **Survey instrument:**questionnaire assessing predictors of acceptance of the   Protectiv® Plus IV catheterdevice.   - **Sampling frame:**fulltime direct patient care registered nurses - **Sampling method:**randomly selected - **Recruitment method:**not reported   **Administration method:**not reported | - **Sample size calculation:**No - **Sampling type:** Probability sampling   **Validity of tool:**Not reported   - **Pilot testing done:**Yes   **Response rate:**83.5%   - **Handling of missing data:** not reported | - **Population:** fulltime   direct patient care registered nurses   - **N:**742 - **Age:**not reported - **Gender:** not reported - **Country:**USA   **Setting:**A 900-bed urban teaching hospital in Texas in 36 nursing units including medical surgical, intensive care, cardiovasculartransplant units, surgical preoperative and post-operative, day surgery, maternal and neonatal units, emergency , oncology clinic and others | - Protectiv® Plus IV | **Individual predictors of acceptance:**  Length of time as licensed nurse in this hospital: The largest group (36.1%) had 10–20 years of experience.  Experience: Almost half of the nurses (48.6%) had used the device more than 12 months.  **Organizational predictors of acceptance:**  Training: the majority of nurses (69.6%) agreed to the appropriateness of time between training and use of device. The majority (76.9%) disagreed that they needed more time for training.  Safety Climate: positive safety climate existed  **Satisfaction:**  **Perceived safety:** 91.6% agreed that proper use of the device protects from needlestick injury  **General satisfaction**: 76.1% of nurses agreed that they are generally satisfied with the device.  **Confidence**: 82.8% of the nurses felt comfortable using the device. 86% responding always use the device. |
| Vaudelle-Malbos, 1996 [[18](#_ENREF_14)]  Funding: not reported  Conflict of interest not reported | - **Type of study:** survey - **Survey instrument:** questionnaire in two sections: first section about ease of use and safety of the device (included binary evaluation yes/no and a 6 point scale); and a second section that asked specific questions about devices. - **Sampling frame**: not reported - **Sampling method:** convenience sampling (departments selected based on utilization patterns) - **Recruitment method**: not reported - **Administration method:** not reported | - **Sample size calculation:** not reported - **Sampling type:** non-probability sampling - **Validity of tool:** questionnaire elaborated with the collaboration of the hygiene supervisor of each hospital; no validation reported - **Pilot testing done**: not reported - **Response rate:**   Safety-Lok: 37%  Needle-Pro: 5%  Protectiv:89%   - **Handling of missing data:** not reported | - **Population: HCWs (nurses in majority, radiology department, physicians) from a range of specialties** - **Number of participants:** varied between 22 and 163 - **Age:** not reported - **Gender:** not reported - **Country:** France - **Setting:** Sainte-Anne hospital | **3 needle protection devices:** Safety-Lok™, Needle Pro®, I.V. catheter Protectiv® and 2 needleless systems (Interlink® and Bionecteu®r) | **Perceived ease of use:** Safety-Lok™ was perceived as very easy to use, but with minor difficulties concerning the insertion of needle and obligation of one attempt.  **Reliability:** Catheter Protectiv®: 8% reported having difficulty to avoid leak of blood after removal of needle from the vein  **Perceived safety:** all devices were perceived as safe except the catheter Protectiv  1/3 of users thought that the Needle-Pro® device was not effective in avoiding needlestick injuries and therefore should not be implemented as a safety device. |
| Casey, 2007[[19](#_ENREF_3)]  Educational grant from Baxter Healthcare corporation  Conflict of interest not reported | - **Type of study:** Interventionfollowed by survey. - **Survey instrument:** 12-month trial of new product followed by distribution of a questionnaire to HCW’s. (Training sessions were provided at the beginning of the trial) - **Sampling frame:** not reported - **Sampling method:** randomly selected - **Recruitment method:** not reported - **Administration method:** not reported | - **Sample size calculation:** not reported - **Sampling type:** probability sampling - **Validity of tool:** not reported - **Pilot testing done:** not reported - **Response rate:** not reported - **Handling of missing data:** not reported | - **Population:** healthcare workers (nursesand anesthetists) - **N:** 40 - **Age**: not reported - **Gender:** not reported - **Country:** UK - **Setting:** City Foundation Trust hospital: In critical care ward and cardiac theatre. | - Needless connector (Clearlink®) | **Perceived safety:** 70% preferred to usethe safer sharps device rather than a conventional luer cap.  **Perceived ease of use:** a positive response was given of more than 85%. **Perceived compatibility:** 85 %considered the device to be suitable for every day practice  **Reliability:** 1 respondent noted a tendency for the device to ‘pop off’ when used with a luer lock syringe **Confidence:** 85% felt confident to use the device after caring for 3 patients |
